# 

**Published:** 1913-03-15

**Authors:** 


### TRIP TO THE RIVIERA FOR EASTER.

The Orient Line announce that on the Friday before Easter week their Australian mail steamer *Orsova*, 12,036 tons, will leave London, and passengers desiring an Easter holiday at sea can proceed by her to Gibraltar and Toulon and return by the *Orvieto*, 12,130 tons, arriving at Plymouth on the Friday after Easter and London the following day. Passengers who disembark at Gibraltar will have five or six days in which to visit Tangier and the South of Spain, while those who proceed to Toulon will have two days on shore in the Riviera. Hyères adjoins Toulon, and Cannes, Nice, and Monte Carlo are within easy reach by rail. The large size and luxurious accommodation of these two vessels should commend this trip to those who know the value of a sea-going holiday. An illustrated programme can be obtained on application to the Orient Line, Fenchurch Avenue or Cockspur Street.

### MODERN POSTER WORK.

Millais, unfortunately the later Millais, was probably the first artist to be captured by an enterprising firm for modern poster work, since when, especially in France, posters have come to be recognised as a department of art as definite as the illustration or the caricature. Everyone remembers the *Peter Pan* poster—Buchell's work, was it not?—and one is glad to see Mr. Joseph Simpson advertising "Oxo" with a poster—which The Hospital has been able to reproduce among its advertisements recently—entitled "Quite well, Doctor; thanks to you and" (of course!) "Oxo." Both the artist and the firm must be congratulated for following the tradition of the mediæval *ateliers*, in which the artist was retained by the prince, the Church, and the merchant to advertise the city, the buildings, and the wares of all three. Has Burlington House a room for posters? If not, it ought to reserve one for this very interesting and alive form of pictorial art.

### The Annual Meeting as an Asset.

We have often pointed out that properly organised and thought-out the annual meeting can frequently be made, at a relatively small expenditure, more fruitful in cash than an annual dinner. Turning over some old records we came across a statement made thirty years ago that the governors of Metropolitan institutions were continuously led to believe that their presence was required rather more at the festival dinner than at the annual meeting. Festival dinners were often a disillusion so far as the results were concerned, and the larger portion of the receipts at them would be obtained by the institution, if efficiently managed, almost without cost. In the case of one London hospital, the committee had very carefully considered this question, and decided to abolish the dinner and boom the annual meeting. The latter was made attractive by securing an eminent chairman and good speakers—that is, the committee substituted real business for fine feeding. In the result seven-tenths of the whole receipts formerly acknowledged at the dinner were sent in in response to an invitation to be present at the annual meeting. The committee further received in new contributions 20 per cent. more than the remaining three-tenths of the average amount formerly yielded by the annual dinner. In this connection we heartily congratulate Mr. P. J. Michelli, who has boomed the *Dreadnought* Hospital annual meeting to be held at Prince's on the 17th inst. The card of invitation might well be framed and preserved. The chairman will be H.S.H. Vice-Admiral Prince Louis of Battenberg, G.C.B.; and the Right Hon. Austen Chamberlain, M.P., and Sir Edward Beauchamp, M.P., Chairman of Lloyds, will attend and speak. Matinées are increasingly popular in the present day, and the *Dreadnought* matinée on the 17th inst. should yield an abundant harvest. Mr. Austen Chamberlain has proved his power in raising money for hospital purposes. When he asked for £50,000, it was sent him so quickly that he increased his appeal to £100,000 in order to spare many people the pain of being too late to have their name placed upon his list.

### Buildings Contemplated and in Progress.

Aberdare.—A committee of the Urban Council has been given powers to complete negotiations with Lord Merthyr for a site for a new isolation hospital in Abernant Park. £8,750 is the sum stated as the cost of the land.

Barrow-in-Furness.—Particulars may be had from the Borough Engineer and Surveyor at the Town Hall for tenders for the erection of two pavilions, laundry, and other works at Devonshire Road Infectious Diseases Hospital.

Birmingham.—Application is to be made by the Guardians for sanction to a loan of £96,000 for additional adult homes and administrative buildings, etc., at Monyhull Colony.

Birmingham.—Messrs. Whitwell and Son, Newhall Street, Birmingham, are architects for a centralised heating and hot-water plant system at Selly Oak House for the Guardians. Tenders are invited.

Bradwell Sanatorium, North Staffs.—Mr. A. G. Bond, B.A., A.R.I.B.A., has given the result of his assessorship of the competition for enlarging Bradwell Sanatorium, and has placed the design by Messrs. A. R. Wood and Son, of Burslem, first, and that by Mr. W. F. Slater, also of Burslem, second. The committee have adopted the assessor's report.

Caerleon.—Pontypool Guardians have approved the purchase by the Unions of the County of a building at Caerleon for the Newport Union as a home for the feeble-minded. The cost is about £7,045.

Canterbury.—The Corporation has approved a proposal to extend the Borough Asylum at an estimated cost of £25,000.

Colchester.—Tenders are invited for the erection of a chapel at Severalls Asylum, Mile End, Colchester. Particulars may be obtained from the quantity surveyors, Messrs. Curtis and Sons, Finsbury Square, London.

Eye.—Hartismere Guardians have adopted plans and estimate of £15,500 for a new infirmary.

Gelligaer.—A new hospital, built at a cost of £12,000 under the supervision of Mr. P. Vivian Jones, architect, Hengoed, has been formally opened.

Gorleston.—Messrs. Olley and Haward, Great Yarmouth, are architects for a home and hospital at Gorleston, and the Guardians have received ten tenders, ranging from £2,499 10s. to £2,791.

Gorleston.—Yarmouth Guardians have decided to borrow £2,678 for a new receiving home for children, and other works.

Kirkham, Lancs.—Mr. F. Harrison, 30 Willow Street, Accrington, is architect for cottage homes at Kirkham for the Fylde Union. Eighteen tenders have been received, ranging from £8,416 to £10,487.

London.—At the annual meeting of the Court of Governors of Middlesex Hospital it was announced that an anonymous donor had expressed his intention of defraying the cost of a new pathological block and institute of hygiene. The cost is estimated at £10,000.

London.—A scheme is under consideration for the provision of further accommodation for chronic cases at Fulham Road Cancer Hospital. Efforts are being made to raise £1,000 for starting a library.

London.—Mr. A. Saxon Snell, F.R.I.B.A., 9 Bentinck Street, Manchester Square, W., is architect for conversions at Marylebone Workhouse, Northumberland Street, W. Particulars may be had for tender from the architect on deposit of a £5 note.

Margate.—Alterations and additions are projected at Margate Cottage Hospital to cost about £12,000.

Portsmouth.—Land in rear of the Infectious Diseases Hospital is to be purchased by the Corporation with a view to immediate future extensions.

Rochdale.—Mr. Hugh Healey, of Rochdale and Manchester, is architect for extensions, etc., at Rochdale Infirmary estimated to cost £30,000.

Ruthin.—Mr. T. A. Roberts, of Mold, architect, has prepared plans for a new infirmary for the Guardians, estimated to cost £4,500.

### A MANY-SIDED LIFE OF ACTION.

Deputy-Inspector-General George Saunders, M.D., C.B., has died at Tunbridge Wells in his ninetieth year. His medical education took place at St. Bartholomew's Hospital and at the Ecole de Médicine, Paris, and after this, in 1845, Dr. Saunders entered the Army as a surgeon. Being quartered in Ireland during the Famine and the disturbances which resulted from it, he devoted himself to relieving the disease which was so prevalent at the time, a depressing task to one who knew that until the cause had been removed there was hardly room for the science of medicine, the only alternative being to see the disease exhaust itself. He saw service in the Crimea, then went to the Cape, and returned to Ireland with medical charge of the depôt at Belfast. He was principal medical officer at Hong Kong in 1865, where he saw another epidemic, this time of fever. His C.B. was the public recognition of his services, the experience of which he laid before the Select Committee on the Mortality of Troops. He retired in 1871, after having declined the post of sanitary officer to the Abyssinian Expedition. Medical Missions, always an interesting subject to him, now engaged his active leisure, and he became director of one in St. Giles, the conditions of life in this then notorious quarter being the subject of some published accounts, which were followed later by his "Reminiscences."

### "THE ARCHITECTURAL REVIEW."

With the New Year "The Architectural Review" assumed a new form in that its appeal, instead of being only to those interested in the fine arts, is now to a wider circle, and a series of articles on "Sanatoria for the Community" are interesting not only to architects but also to those who have to do with the development and management of such institutions. The introduction to the series in the January number briefly summed up the present position in respect to sanatoria, and was followed by an article from the pen of Mr. Theodore Fisher, of Shadwell Children's Hospital. Mr. J. E. Drower contributed "Some Fundamental Considerations" from his experience of Swiss sanatoria, and "Benenden Sanatorium" was described and illustrated by Mr. A. William West, architect; it will be remembered that he was with Dr. Latham joint author of the King Edward Prize Essay on Sanatoria. An example of the tent system from Colorado Springs, U.S.A., by Messrs. McLaren and Thomas, is also illustrated and described. The February number contained an article on "The Experience of Germany," by Mr. C. L. Leipoldt, F.R.C.S., which dealt largely with the aspect of cost, and concludes by stating that no sanatorium in this country need cost more than a maximum of £200 per bed for substantial buildings, and further suggests that tuberculosis camps on the lines of the North Carolina system might be followed in this country at a cost of a few pounds per patient.

### Mount Vernon Hospital.

As we go to press we learn that at the annual meeting on the 12th inst. the resolution regarding the sale of the property at Hampstead was carried with two dissentients only. That resolution, proposed by Mr. Charles Johnston and seconded by Mr. Basil Lawrence, empowers the committee of management to submit the hospital situated at Hampstead for sale by public auction or otherwise, and to take the necessary steps to effect such sale.

### Appointments.

THE FIRST PROFESSOR OF PHARMACEUTICS.

The Pharmaceutical Society has received from the University of London resolutions of the Senate conferring the title of University Professor on Dr. A. W. Crossley and Mr. H. G. Greenish, the two professors of the Society's School of Pharmacy. Professor Crossley, D.Sc., Ph.D., F.R.S., has been Professor of Chemistry in the Pharmaceutical Society's School since 1904. Professor Greenish, F.I.C., F.L.S., is Dean of the School, and since 1896 has been Professor of Pharmaceutics. He is the first Professor of Pharmaceutics to be recognised by the University.

ARMY MEDICAL ADVISORY BOARD.

The Secretary of the War Office announces that the Secretary of State for War has approved the appointment of Sir Anthony A. Bowlby, C.M.G., F.R.C.S., to be Civilian Member of the Army Medical Advisory Board, as Surgeon-General Sir R. Havelock Charles, G.C.V.O., will in future represent the India Office on the Board.

Dr. James Mackenzie is to be appointed in charge of the new department for the study of heart disease and rheumatism to be established at the London Hospital.

Mr. Frank J. Wethered, M.D., F.R.C.P., has been appointed fourth physician and Mr. Edward Alfred Cockayne, M.D. (Oxon.), M.R.C.P., previously medical registrar, an assistant physician to the Middlesex Hospital.

Dr. W. C. Bosanquet, formerly assistant physician, has been appointed full physician at the Brompton Hospital for Consumption in place of Dr. H. W. Mackenzie, who has resigned; and Dr. L. S. T. Burrell has been elected assistant physician in the vacancy.

### Gifts and Bequests.

LARGE BEQUEST TO AMERICAN HOSPITALS.

Mr. John Torrance Vanneck, of Manhattan, New York, has left $50,000 to the Montreal General Hospital and $50,000 to the New York Post-Graduate Medical School and Hospital, Manhattan, as an addition to the endowment.

A HOSPITAL CHAIRMAN'S WILL.

The Rev. Arthur Brinckman, lately chairman of St. Saviour's Hospital, N.W., and formerly an officer in the Army, has left net personalty £20,291. He bequeathed £4,000 for the benefit of St. Saviour's Hospital. Subject to numerous other bequests to relations, nurses, and others, he left the residue of his property equally between three charities, including the Actors' Orphanage Fund.

A DOCTOR'S BEQUESTS TO SERVANTS.

Mr. Duncan Macdonald Forbes, F.R.C.S., L.R.C.P., medical officer of health for the Eastwood (Notts) Urban District Council, left net personalty sworn at £78,344. The testator left £4,000 and a life annuity of £150 to his valet, £1,000 and a life annuity of £100 to his coachman, a life annuity of £80 to his cook, legacies to other servants, £10,000, £4,000, and £3,000 to three solicitors, and bequests to relatives, and, subject to his wife's life interest, £1,000 to the Nottingham General Hospital and £500 to the Eastwood Nursing Association.

By his will, it is stated, the testator had left £5,000 to the Nottingham General Hospital, £2,000 to the Nottingham Children's Hospital, and £8,000 for almshouses in the district, but he revoked these bequests by codicil.

Miss Dains, of Ipswich, left by her will £500 each to Dr. Barnardo's Homes, Fegan's Homes, Deptford, Spurgeon's Stockwell Orphanage, and the Eastern Counties Asylum for Idiots at Colchester.

Sir Gilbert Wills, M.P., has offered £5,000 in memory of the late Sir Frederick Wills to the Royal Victoria and West Hants Hospital, Bournemouth, towards the building of a long-hoped-for administration block at the Boscombe branch. Sir Frederick Wills for many years was president of the Royal Boscombe and West Hants Hospital, and after its amalgamation with the Royal Victoria Hospital, Bournemouth, Sir Gilbert Wills was elected as joint president with Lord Malmesbury.

The Pontypool and District Hospital has received from Mr. Alfred Addams Williams a share certificate for £100 invested in the Pontypool Gas and Water Co. Mr. Williams has also been good enough to give them the interest on the share. Although through circumstances of age and removal, Mr. Williams could not attend their meetings now, his interest, it is felt, is as lively and warm to-day as ever it was. He was the chairman of the committee which inaugurated the hospital, and was able to declare it open free of debt, a gratifying event largely the result of his efforts.

### KING EDWARD'S HOSPITAL FUND FOR LONDON.

We are asked to state that hospitals in the County of London, or within nine miles of Charing Cross, which wish to participate in the grants made by this Fund for 1913 must make application before the 31st instant to the Honorary Secretaries, 7 Walbrook, E.C. Applications will also be considered from convalescent homes which are within the boundaries mentioned, or which, being outside, take a large proportion of patients from London; and from sanatoria for consumption which take patients from London, or which are prepared to place beds at the disposal of the Fund for the use of patients from London hospitals.

### THE PROSPERITY OF THE "PRUDENTIAL."

The sixty-fourth annual report of the Prudential Assurance Company, Limited, repeats what the public has come to regard as its customary record of prosperity. The number of policies in force in the ordinary branch at the end of the year was 901,838. "Arising out of the National Insurance Act the directors found there was a demand for certain additional and voluntary sickness insurances supplemental to the insurances afforded by the Act." In more senses than one, in fact, the National Insurance Act appears to have increased the business and added to the prosperity of this vast insurance corporation.

